# Rhabdomyosarcoma Cells Produce Their Own Extracellular Matrix With Minimal Involvement of Cancer-Associated Fibroblasts: A Preliminary Study

**DOI:** 10.3389/fonc.2020.600980

**Published:** 2021-01-29

**Authors:** Stefania D’Agostino, Lucia Tombolan, Mattia Saggioro, Chiara Frasson, Elena Rampazzo, Stefania Pellegrini, Francesca Favaretto, Carlo Biz, Pietro Ruggieri, Piergiorgio Gamba, Paolo Bonvini, Sanja Aveic, Roberto Giovannoni, Michela Pozzobon

**Affiliations:** ^1^ Stem Cells and Regenerative Medicine Lab, Fondazione Istituto di Ricerca Pediatrica Città della Speranza, Padova, Italy; ^2^ Department of Women and Children Health, University of Padova, Padova, Italy; ^3^ Pediatric Solid Tumors Laboratory, Fondazione Istituto di Ricerca Pediatrica Città della Speranza, Padova, Italy; ^4^ Onco-Hematology Laboratory, Fondazione Istituto di Ricerca Pediatrica Città della Speranza, Padova, Italy; ^5^ Brain Tumors Laboratory, Fondazione Istituto di Ricerca Pediatrica Città della Speranza, Padova, Italy; ^6^ Department of Medicine, Internal Medicine 3, University of Padua, Padua, Italy; ^7^ Department of Surgery, Oncology and Gastroenterology DiSCOG, Orthopedic Clinic, University of Padova, Padova, Italy; ^8^ Neuroblastoma Laboratory, Fondazione Istituto di Ricerca Pediatrica Città della Speranza, Padova, Italy; ^9^ Department of Dental Materials and Biomaterials Research, Rheinisch-Westfälische Technische Hochschule (RWTH) Aachen University Hospital, Aachen, Germany; ^10^ Department of Biology, University of Pisa, Pisa, Italy

**Keywords:** rhabdomyosarcoma, cancer-associated fibroblasts, tumor microenvironment, extracellular matrix proteins, stroma

## Abstract

**Background:**

The interplay between neoplastic cells and surrounding extracellular matrix (ECM) is one of the determinant elements for cancer growth. The remodeling of the ECM by cancer-associated fibroblasts (CAFs) shapes tumor microenvironment by depositing and digesting ECM proteins, hence promoting tumor growth and invasion. While for epithelial tumors CAFs are well characterized, little is known about the stroma composition of mesenchymal cancers, such as in rhabdomyosarcoma (RMS), the most common soft tissue sarcoma during childhood and adolescence. The aim of this work is to identify the importance of CAFs in specifying RMS microenvironment and the role of these stromal cells in RMS growth.

**Methods:**

We assessed in two dimensional (2D) and three dimensional (3D) systems the attraction between RMS cells and fibroblasts using epithelial colon cancer cell line as control. CAFs were studied in a xenogeneic mouse model of both tumor types and characterized in terms of fibroblast activation protein (FAP), mouse PDGFR expression, metalloproteases activation, and ECM gene and protein expression profiling.

**Results:**

In 2D model, the rate of interaction between stromal and malignant cells was significantly lower in RMS with respect to colon cancer. Particularly, in 3D system, RMS spheroids tended to dismantle the compact aggregate when grown on the layer of stromal cells. *In vivo*, despite the well-formed tumor mass, murine CAFs were found in low percentage in RMS xenogeneic samples.

**Conclusions:**

Our findings support the evidence that, differently from epithelial cancers, RMS cells are directly involved in their own ECM remodeling, and less dependent on CAFs support for cancer cell growth.

## Introduction

Rhabdomyosarcoma (RMS) represents approximately 4% of all cancers diagnosed during childhood and adolescence (1). With an incidence of 4.5 cases per million of youth population per year, RMS is the most common soft tissue sarcoma (2). The onset of RMS has been attributed mainly to the cells of myogenic lineage but more recently also non-myogenic mesenchymal cells were indicated as possible RMS progenitors (3, 4). As a result, RMS can develop not only in skeletal muscles, like head and neck, trunk, and extremities, but also in distant sites, including genitourinary and biliary tract. The most common histological subtypes are embryonal (ERMS), with favorable prognosis, and alveolar (ARMS) RMS, with worse patient outcome (5). The multimodal therapy that combines surgical resection, radiation and chemotherapy has been adopted over the past 3 decades for the cure of patients with RMS. This therapy regimen significantly improved the survival rate of the patients with localized disease. Yet, overall survival of RMS patients remains poor when metastases occur, particularly in the bone marrow, lungs, and lymph nodes (1). Cancer spreading and metastasis formation is a complex mechanism that requires multiple players to be successful, including many components of the surrounding tumor microenvironment (TME), both cellular and acellular. Indeed, while healthy tissue and stroma share tumor suppressive features (6), in pathological conditions substantial changes occur and the environment adapts to the needs of tumor cells, supporting their survival and proliferation (7–9).

The TME is composed of the extracellular matrix (ECM), built on both non-cellular (proteins, glycosaminoglycans, growth factors) and cellular components (e.g. immune cells, fibroblasts, endothelial cells). Together, cellular and non-cellular components of the TME determine the aggressiveness and the pro-metastatic potential of tumor cells. Fibroblasts, instead, are the major stromal cell population and are critical determinants of cancer cell-stroma crosstalk. In healthy tissues, fibroblasts are responsible for ECM proteins and matrix metalloproteases (MMPs) production, acting as tumor suppressors (10). During wound healing process, they acquire an activated phenotype and increase the deposition of ECM proteins. Under these circumstances, fibroblasts express alpha-smooth muscle actin (α-SMA) protein, becoming myofibroblasts (11), and drive wound contraction thanks to the strong inner actomyosin forces (12).

In cancer, the permanent activated phenotype of fibroblasts give rise to cancer-associated fibroblasts (CAFs), and due to such behavior, cancer is usually considered a “wound that does not heal” (13). The role of CAFs is mainly defined in epithelial tumors and it is focused on the modification of the ECM protein deposition: fibrillar collagens, type I and III are increased as well as the amount of glycoproteins, including fibronectin, laminin, elastin, and proteoglycans (14). These microenvironment changes contribute to the formation of the metastatic niche. In addition, the deposition of ECM proteins is accompanied by activation of ECM-degrading matrix metalloproteinases (MMPs) enzymes which represents a driving event for fibroblasts activation, cancer cells migration and invasion (15). CAFs are heterogeneous and different subsets are found in one single tumor (16, 17). Among all, one of the classical CAF markers is the fibroblast activation protein (FAP), a serine protease with a gelatinase domain (18). FAP activity is paramount to sustain CAFs growth and TME development, likewise cancer cell survival, invasive cell properties, and neoangiogenesis (17). FAP is particularly expressed by normal fibroblasts that turn into CAFs (19, 20). These activated fibroblasts are present in many epithelial tumors (colorectal cancer, breast carcinoma), but in tumors of mesenchymal origins, such as RMS, have so far only started to be investigated (21, 22).

To date, little is known about TME composition in RMS tumors and CAFs role in pathological ECM remodeling (21, 23). In order to better understand the cell-TME interaction, three-dimensional (3D) models have been developed since the flat two-dimensional (2D) cell cultures are too far to mimic the complex physiological interconnections that happen in patients (24). In this preliminary study, using 2D *in vitro* and 3D models (spheroids and *in vivo* xenogeneic samples), we assessed the RMS cells invasiveness characteristics, in term of ECM proteins expression and the contribution that CAFs have in determining ECM composition, dictating RMS tumor cell growth and behavior.

## Materials and Methods

### Cells

The RH30 (ARMS) (RRID : CVCL_0041), RD (ERMS), and MCF7 (breast cancer) (RRID : CVCL_0031) cell lines were kindly provided by the Solid Tumours lab (Prof. Bisogno, Padova, Italy), HT29 (colon carcinoma cells) were kindly provided by Nano Inspired Lab (Dr. Agostini, Padova, Italy). RH30 were stably transduced with pRRLsin.PPTs.hCMV.GFPpre vector to obtain GFP-expressing RH30 (GFP^+^ RH30). BJ healthy skin fibroblasts were kindly provided by Dr. Radu, Department of Women and Children Health, Padova, Italy. All cell lines were cultured in high glucose DMEM, supplemented with 10% fetal bovine serum (FBS, Gibco), 1% penicillin/streptomycin, 1% L-glutamine (all reagents were from Gibco, Monza, Italy) in tissue culture flasks (Sarstedt, Verona, Italy) at 37°C, 5% CO2, and 95% relative humidity.

### Primary Cells

Primary human muscle precursor cells (hMPC) were isolated from discarded muscle biopsy (protocol number P3030 and 2682P Azienda Ospedaliera of Padova), following the protocol described in (25). Wharton’s jelly-derived mesenchymal stromal cells (MSC) were provided by Esperite—The cell factory, Niel, Belgium. Cells were cultured in high glucose DMEM, supplemented with 10% FBS, 1% penicillin/streptomycin, 1% L-glutamine (all reagents were from Gibco, Monza, Italy). Pictures of all cells were taken using an Olympus IX71 microscope.

### Animals

In this work only xenogeneic tumor samples have been used. Twelve-week-old male and female (C;129S4-*Rag2^tm1.Flv^ Il2rg^tm1.Flv^/J*, also called *Rag2^−/−^γc^−/−^*) mice were used as recipients for subcutaneous flank injections for xenograft production following three different procedures: (1) the classical, single cell type resuspended in Matrigel^®^ (Corning, Tewksbury, USA): 2 × 10^6^ RMS cells or 5 × 10^6^ colon carcinoma or breast cancer cells (10 mice for RH30, 10 for RD and 10 for HT29 cells, 6 for MCF7 cells); (2) tumor cells together with the fibroblasts (without Matrigel^®^): 1 × 10^6^ RMS cells or 2·5 × 10^6^ colon carcinoma with 1 × 10^6^ and 2·5 × 10^6^ BJ cells (10 mice for RH30, 10 for RD, and 10 for HT29 cells); (3) single cell type without Matrigel^®^ or other cell support: 2 × 10^6^ RMS cells or 5 × 10^6^ colon carcinoma cells. The treatment was approved by Animals care and Use Committee (CEASA, protocol 304/2017) and were communicated to the Ministry of Health and local authorities in accordance with the Italian Law (DL n. 16/92 art. 5). Xenogeneic samples were harvested 21 days post injection for RMS cells [for characterization of RMS samples see (26)] and 15 days post injection for colon (HT29) and breast carcinoma (MCF7) cells. Four mice per group were used for cell sorting. Xenografts were weighted and the volume was evaluated multiplying height × width × length using caliper.

### Cell Migration Tests

Migration rate was assessed using 8 µm-pore transwell inserts (Sarstedt, Verona, Italy) in a 24-well plate: 1 × 10^5^ cancer cells (or fibroblasts) were seeded in the upper chamber, and 5 × 10^4^ fibroblasts (or cancer cells) were seeded in the lower chamber. After 24 h, membranes were fixed in 4% PFA (Sigma-Aldrich, Saint Louis, USA) and stained with Hoechst (Life Technologies, Carlsbad, USA), for 15 min. They were then dehydrated in ascending alcohol series and transferred on glass slides using Eukitt^®^ quick-hardening mounting medium (Sigma-Aldrich, Saint Louis, USA) and a coverslip. For each cell line six independent experiments in triplicate were assessed. For each transwell, 10 random pictures, 10× magnification were taken using Leica DMI6000B microscope, collected and analyzed.

### Spheroids

The RH30, GFP^+^ RH30, and RD spheroids were obtained seeding 5 × 10^3^ cells in ultralow-adhesion, round-bottom 96-well plates (Corning, Tewksbury, USA) in low glucose DMEM supplemented with B27 (both from Gibco, Monza, Italy), 10 ng/ml bFGF and 20 ng/ml EGF (both from ORF Genetics, Kopavogur, Iceland). HT29 spheroids were produced seeding 5 × 10^3^ cells in the same plate in high glucose DMEM (Gibco, Monza, Italy) supplemented with 5% FBS (Gibco, Monza, Italy). After 5 days, cell viability was assessed using Cell Titer-Glo Luminescent assay kit (Promega, Madison, USA). Five days after seeding, each spheroid was transferred on a layer of 1 × 10^4^ BJ or MSC. These cells were used as stromal layer and the spheroid-stromal interaction was monitored for 48 h using Leica DMI6000B microscope. Cytotoxicity was analyzed with the Lactate Dehydrogenase Activity (LDH) Assay Kit (Sigma-Aldrich, Saint Louis, USA). Supernatant of stromal cells (BJ and MSC) and of spheroids alone was used as control. Absorbance was expressed as fold change in respect to the control. Alive cells were detected with Live and Dead staining (Thermo Fisher, Waltham, USA) following manufacturer’s instructions. Five experiments were performed for each condition.

### Xenograft Processing

Xenotransplants were minced with sterile blades, digested using 0.2% Collagenase II, 1 mg/ml Collagenase IV (Sigma-Aldrich, Saint Louis, USA) at 37°C for 90 min and then with trypsin (Gibco, Monza, Italy) for 90 min at 37°C. The digestion product was filtered using 70 or 40 µm cell strainers and stained for 15 min at RT with the antibodies listed in **Table 1**. Analyses were performed using Accuri BD cytofluorimeter or sorted. Specifically, 3–5 × 10^6^ cells/ml were incubated with anti-mouse mPDGFRα (APC conjugated, Becton and Dickinson) and analyzed on a MoFlo High Performance Cell Sorter (Beckman Coulter). mPDGFRα^+^ and mPDGFRα^−^ cell fractions were selected and sorted on the basis of mPDGFRα expression. Relative percentages of the two different subpopulations were calculated based on live gated cells (as indicated by physical parameters, side scatter and forward scatter, and 7AAD negative cells) (7AAD is provided by Invitrogen, Carlsbad, USA). After sorting, an aliquot of the sorted cells was run on the MoFlo to check the purity of the two populations. The reached overage purity was higher than 92% (93 ± 1.2% of purity for the positive fraction and 98 ± 0.5% of purity for the negative fraction).

**Table 1:** Antibody list for cytofluorimetric analysis.

Antibodies, Company	Fluorophore
Anti-human CD140A or PDGFRα (BD biosciences, San Jose, USA) (RRID : AB_396286)	PE
Anti-murine CD140A or PDGFRα (BD biosciences, San Jose, USA) (RRID : AB_2737788)	APC
Anti-CD184 or CXCR4 (BD biosciences, San Jose, USA) (RRID : AB_396267)	PE
Anti-CD44 (Thermo Fisher Scientific, Waltham, USA) (RRID : AB_465045)	FITC
Anti CD73 (BD biosciences, San Jose, USA) (RRID : AB_393561)	PE
Anti-CD90 (BioLegend, San Diego, USA) (RRID : AB_893429)	FITC
Anti-CD105 (Beckman Coulter, Brea, USA) (RRID : AB_1575944)	PE

### Immunofluorescence

Ten samples from xenografts and from human muscle tissue (used as control, Ethical protocol number P3030 and 2682P Azienda Ospedaliera of Padova) were fixed in 4% PFA and included in Killik cryostat embedding medium (Bio-Optica, Milano, Italy). Samples were stored at −80°C until they were cut on 7 μm slices using Leica CM1520 cryostat. For immunofluorescence analyses, samples were incubated with primary antibodies overnight at 4°C and later with secondary antibodies Alexa Fluor-conjugated. Antibodies used are listed in **Table 2**. Nuclei were counterstained with fluorescent mounting medium plus 100 ng/ml 4’,6-diamidino-2-phenylindole (DAPI) (Sigma-Aldrich, Saint Louis, USA). Each sample was prepared carrying 7 μm tissue slides (n = 10), cutting the whole xenograft, from the border to the center of the sample. The same procedure was used for human healthy muscles. For each count performed, 10 random pictures at 20× magnification, were collected and analyzed.

**Table 2:** Immunofluorescence antibody list.

Antibodies, Company	Dilution	Incubation time
Rabbit anti-FAP (Abcam, Cambridge, UK) (RRID : AB_880077)	1:50	Overnight at 4°C
Mouse anti-fibronectin (Invitrogen, Carlsbad, USA) (RRID : AB_10982280)	1:100	Overnight at 4°C
Mouse anti-Human Nuclei (Millipore, Burlington, USA) (RRID : AB_94090)	1:200	1 h at RT
Mouse anti-MYOD (Agilent, Santa Clara, USA) (RRID : AB_2148874)	1:50	1 h at RT
Rabbit anti-laminin (Sigma-Aldrich, Saint Louis, USA) (RRID : AB_477163)	1:200	1 h at 37°C
Goat anti-Mouse IgG Secondary Antibody, Alexa Fluor 488 (Invitrogen, Carlsbad, USA) (RRID : AB_2534069)	1:200	1 h at 37°C
Chicken anti-Rabbit IgG Secondary Antibody, Alexa Fluor 488 (Invitrogen, Carlsbad, USA) (RRID : AB_141735)	1:200	1 h at 37°C
Goat anti-Mouse IgG Secondary Antibody, Alexa Fluor 594 (Invitrogen, Carlsbad, USA) (RRID : AB_141372)	1:200	1 h at RT
Chicken anti-Rabbit IgG Secondary Antibody, Alexa Fluor 594 (Invitrogen, Carlsbad, USA) (RRID : AB_141840)	1:200	1 h at RT

### Zymography

Total of 5 × 10^4^ cells from 2D cultured cell lines and xenogeneic-derived cells were seeded in six-well plate in 1·5 ml serum-free DMEM (27) The serum-free conditioned medium was harvested after 24 h for zymography. Zymography was carried out as previously described (28). MMP-2 and 9 quantification was performed using Fiji software.

### Real-Time Quantitative PCR (qPCR)

Total RNA was extracted from 2D adherent cell lines, xenogeneic samples-derived cells and human muscle samples using RNeasy Plus Mini kit (QIAGEN GmbH) or Trizol reagent (Life Technologies) following the supplier’s instructions. RNA was quantified with a ND-2000 spectrophotometer. For all the samples 1 µg of total RNA was reverse transcribed with SuperScript II (Life Technologies) in a 10 µl reaction, while 20 ng of total RNA purified from sorted cells was retrotranscribed with SuperScript III following supplier’s instructions. Real-Time PCR reactions were performed using a Viia 7 Real-Time PCR System (Applied Biosystem); reactions were carried out in triplicate using SYBR Green master mix (Applied Biosystem) and primer mix (final concentration, 200 nM) in a final reaction volume of 10 µl. A relative quantification (RQ) was calculated by ΔΔCt methods using a software implemented in Viia7 Real-Time PCR System. *GAPDH* was used as reference gene for normalization and fetal skeletal muscle or HT29 cells were used as calibrator sample. For each quantification, a confidence interval (IC) of 95% was calculated. Primer sequences used are listed in **Table 3**, and all primers amplified both human and murine sequences. All graphs displayed were produced with GraphPad software 6. Data are displayed as means ± IC 95% (confidence interval, IC).

**Table 3:** Primer list.

GENE	SEQUENCE	Tm	NM
*GAPDH-F*	TCCTCTGACTTCAACAGCGA	60°C	NM_001256799.3
*GAPDH-R*	GGGTCTTACTCCTTGGAGGC		
*FN1-F*	GAAGACATACCACGTAGGAGAACA	57°C	NM_001306129.2
*FN1-R*	AGGTCTGCGGCAGTTGTC		
*MMP2-F*	CAGGAGGAGAAGGCTGTGTT	60°C	NM_001127891.2
*MMP2-R*	GGTCAGTGGCTTGGGGTA		

### Statistical Analysis

Image based counts and measurements were performed with Fiji (29). For each analysis, at least five random pictures were used for data output. All graphs displayed were produced with GraphPad software 6. Data are expressed as means ± SD. For all experiments (qPCR and tissue analysis), statistical significance was determined using an equal-variance Student’s t test or Mann–Whitney U test to compare two groups (i.e. hMT *vs* RH30, HT29 *vs* RH30 or HT29 *vs* RD). Kruskal-Wallis test was applied to compare all groups. A p value below 0.05 was considered to be statistically significant.

## Results

### 2D Cell Monolayers and 3D Spheroids of Rhabdomyosarcoma Do Not Attract Fibroblasts

**Figure 1:**
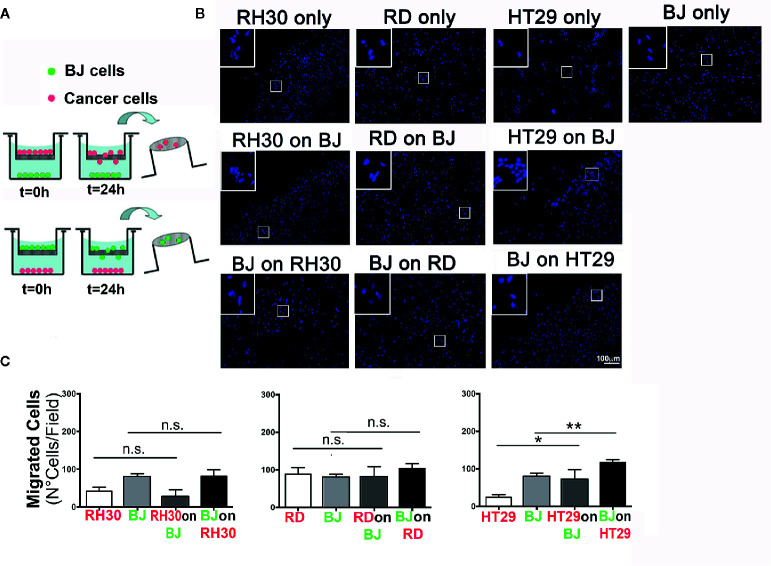
Migration assay. **(A)** Schematic representation of the transwell procedure. Initial experimental conditions (T = 0 h) and data evaluation (T = 24 h) were depicted for RMS (RH30 and RD cells) and CRC (HT29) cells. **(B)** Representative images (10× magnification) of the migrated cells are shown. Insets indicate higher magnification (20×) of the cells that crossed through the porous membrane. DAPI (blue) marks cell nuclei. Scale bar = 100 µm. **(C)** Number of cells per field that passed through the membrane was determined for mono-culture (control RH30, RD, BJ, and HT29 only) or double cell combinations (“on,” x axis of the graph, indicates the cell line grown on top of transwell porous membrane; lower positioned cell line was grown inside the well plate); *p = 0.01, **p < 0.01; n.s., not significant.

In order to evaluate the possible interactions between RMS cells and the stromal microenvironment, we analyzed the ability of different tumor types and fibroblast to crosstalk. To this extent, we examined the ability of RH30 (ARMS), and RD (ERMS) cells to interact with BJ fibroblasts (**Figures 1A, B**). As a positive control we used HT29 cells of colorectal cancer (CRC) origin, an epithelial carcinoma in which activated fibroblasts play a pivotal role in TME remodeling (30). With the classical migration transwell assay, we tested the migration ability of the cancer cells seeded on the top of transwell toward the monolayer of BJ. We studied also the migration of the fibroblasts BJ (on the top of the transwell) toward the RMS and colorectal cancer cells (seeded at the bottom of the well). In addition, the three types of cancer cells alone and BJ, alone, were seeded on the top of the transwell without any other cell type at the bottom of the well, in order to count the cells migrated without any external stimuli (control) (**Figure 1A**). In these settings, RMS RH30 and RD cells were not attracted by BJ cells, and *vice versa*, as none of the two cell lines significantly migrated toward the other unlike it was observed with HT29 and BJ cells (**Figures 1B, C**). This finding confirmed that the stromal cells were prone to migrate toward the HT29 cell line and suggested the lack of a crosstalk with RMS cells (**Figure 1B**).

**Figure 2:**
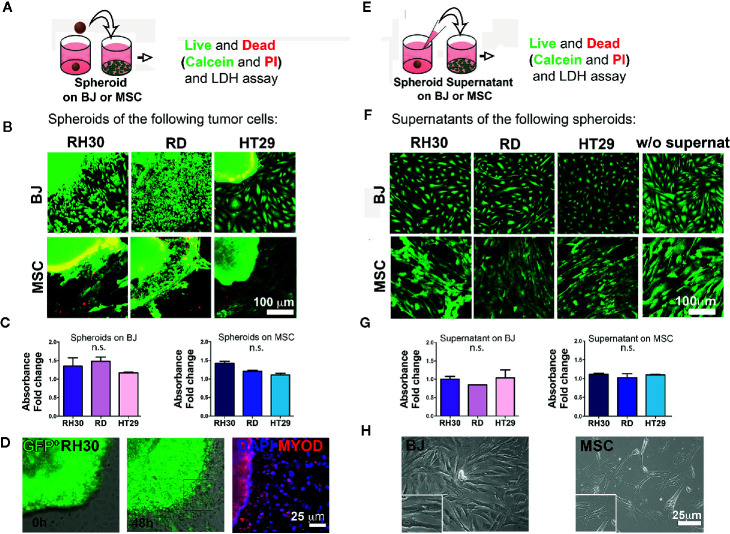
Cancer cells-stroma interaction. **(A)** Cancer cell spheroids were added on top of stromal cells (BJ or MSC) monolayer and live and dead assay was performed after 48 h. **(B)** Cancer cell derived spheroids were added on 2D grown stromal cells (BJ or MSC) and live and dead assay was performed. Calcein (green)-alive cells; PI (red)-dead cells. The yellow color is the effect of the green/red overlapping. Both RH30 and HT29 cells stained negative for PI suggesting no occurrence of cell death. Bright yellow fluorescence for BJ-HT29 and MSC-RH30 images was due to image background signal in red. Scale bar = 100 µm. **(C)** LDH assay. BJ and MSC were cultured with spheroids for 48 h and LDH was measured. Absorbance fold change was calculated relatively to the control (BJ or MSC alone. Set value: 1). Both Kruskal-Wallis and Mann-Whitney tests were used to confirm the lack of toxicity. **(D)** GFP^+^ RH30 spheroids disperse their cells when in contact with BJ. MYOD positive staining demonstrate how cells migrate from the spheroid. **(E)** Supernatants of cancer cell spheroids were added on top of stromal cells (BJ or MSC) monolayer and live and dead assay was performed after 48 h. **(F)** Live and dead assay [calcein—green; and propidium iodide (PI) in red] in BJ and MSC cultured with the different spheroid supernatants and without supernatants. No death events (red signal) were found. **(G)** LDH assay. BJ and MSC were cultured with different supernatants for 48 h and LHD was measured. Absence of supernatant toxicity was confirmed by both Kruskal-Wallis and Mann-Whitney statistical tests. **(H)** Phase contrast microscopy show the morphology of the BJ and MSC after supernatant conditioning. n.s., not significant.

To better investigate the complexity of the cell-cell interactions between RMS and stroma cells, we moved from the 2D assay to the production of 3D spheroids from different cell lines. At first, an optimal cell number and time window for the spheroid formation was defined and in **Figure S1** can be appreciated the round shape and the good viability after 5 days of culture. According to previously established protocols (31), over the cultured monolayer of stromal cells (BJ or mesenchymal stromal cells—MSC), either a single spheroid or RMS-conditioned medium was added (**Figures 2A, E**) and cell viability was assessed. At first, we detected the presence of the classical mesenchymal stromal surface makers (CD44, CD73, CD90, CD105) in BJ cell line and in the heterogeneous primary cells MSC (32–34) (**Figure S2**). Secondly, we observed that the well-defined RH30 and RD spheroid edges disaggregated when in contact with BJ or MSC stromal cells (**Figures S3B** and **C**), most likely due to spreading of calcein stained RMS cells in **Figure 2B**, along with BJ displacement. The spheroids cultured alone were well shaped, compact, and alive (**Figure S3A**). The cells positive for MYOD, myogenic marker for RH30 and RD cells, migrated outside the spheroids. This was previously observed with spheroids of GFP+ RH30 on BJ (**Figure 2D**). The edges of HT29-derived spheroids, instead, remained well defined when layered on both BJ and MSC cells (**Figures 2B** and **S3B**). With respect to cell toxicity, we measured LDH release in the growth medium of stromal cells grown in the presence or absence of cancer spheroids. The amount of LDH release was not statistically significant, when both HT29 and RMS spheroids were put on stromal cells. This supports the concept that RMS cells displace stromal cells, most likely overwhelming their growth without perturbing the cell viability (**Figure 2C**). Migration toward the periphery of both RH30 and BJ cell lines was observed when the cells were grown together (**Figure S3**). We assessed whether contact-dependent or -independent mechanisms were responsible for such behavior. Therefore, as first step, we investigated whether paracrine factors could dictate such behavior. We used RMS conditioned medium in order to investigate whether the secretion of molecules from tumor cells could influence the crosstalk between the two subpopulations. To this intent, BJ and MSC stromal cells were grown in the presence or absence of spheroid-conditioned medium from both RMS and colon cancer cells (**Figure 2E**), and viability was assessed as previously described. As expected, Calcein/Propidium Iodide (PI) staining highlighted alive cells in both BJ and MSC stromal cells grown in the spheroid-conditioned medium (**Figure 2F**), further sustained by the absence of LDH release and changes in cell morphology (**Figures 2G** and **H**, respectively). This was also confirmed for spheroids alone (**Figure S3A**). Although this aspect merits further investigation, it is important to underline that such influence of RH30 cancer cells on BJ fibroblasts migration (and *vice versa*) was not observed when BJ cells were cultured in the presence of HT29 colon cancer cells.

### Lack of CAFs Recruitment Was Observed in RMS Xenogeneic Samples

*In vitro* we demonstrated that RMS cells are not attracted to stromal cells (when not in contact), we then assessed the same phenomenon *in vivo*. At first, we tried the orthotopic implant of RMS cells (**Figure S4**), but the growth of tumor together with the healthy muscle made the tissue and the cell analysis confusing (the murine healthy muscle grew wrapped with the human tumor mass making the tissue morphology and the cell composition cumbersome to be analyzed). Consequently, we employed three different experimental approaches using subcutaneous injection. For all the three experimental strategies the histology was comparable and the similarity between our xenografts (**Figure S5**) and the human sample biopsies can be appreciated comparing **Figure S5** and the images in PathologyOutlines.com (35).

**Figure 3:**
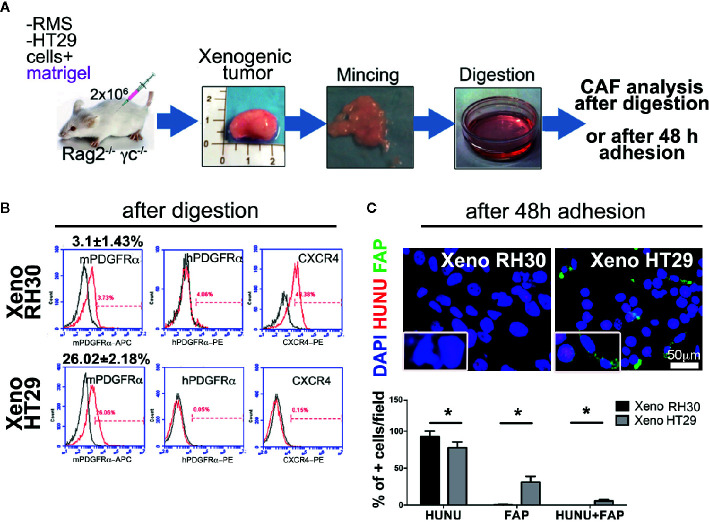
Isolation and characterization of CAFs in xenogeneic samples. **(A)** Experimental strategy (1): cancer cells are subcutaneously injected with Matrigel^®^. Cartoon of the entire *in vivo*/*ex vivo* experimental procedure is presented. Tumors were harvested, digested, and cell immunophenotype were analyzed in following. **(B)** Flow cytometry analysis for the expression of murine or human PDGFRα (mPDGFRα, hPDGFRα, respectively), and the invasion marker CXCR4. p value of RH30 mPDGFRα *versus* HT29 mPDGFRα = 0.0005. p value of RH30 CXCR4 *versus* HT29 CXCR4 = 0.0007. hPDGFRα in RH30 4% ± 0.9; hPDGFRα in HT29 0.05% ± 0.01. **(C)** Upper row. Cells extracted from the RH30 xenogeneic masses were grown in culture for 48 h, and subsequently analyzed for the fibroblasts activation protein (FAP; green) and for human nuclei (HUNU; red) expression. DAPI was used for nuclei counterstaining. Insets represents a closer overview of positive cells for each of the markers. Lower row. Graph bars present a percentage (%) of positive cells respect to total cell nuclei counted per field. Only cells from epithelial masses expressed FAP. Scale bar = 50 µm, *p = 0.05.

Regarding the first approach, RH30, RD, or HT29 cell lines were injected with biomimetic ECM (Matrigel^®^), as the epithelial breast cancer MCF7 cells were, included in the study to validate RMS model in our immunocompromised mice (**Figure S6**). Indeed, while it is well known that epithelial tumors recruit CAFs to grow in nude mice (36), it was important to demonstrate that this also happen in Rag2*^−^* ^/^ *^−^* γc*^−^* ^/^ *^−^* mice. Furthermore, the CAF presence has only started to be investigated for RMS tumors (22). After obtaining single-cell suspension from explanted tumor masses (**Figures 3A** and **S5**), cytofluorimetric analysis of CAFs marker PDGFRα, of both, human and murine origin, was performed. Xenogeneic RMS tumors recruited ten times less mPDGFRα-positive cells than those found in HT29 and MCF7 xenografts, providing two-fold evidence: firstly, our mouse model validated the importance of CAFs in sustaining epithelial cancers formation, and secondly, we start underlining the CAFs dispensability in RMS tumor onsets (**Figures 3A, B**). Interestingly, the human PDGFRα in RMS, HT29, and MCF7 cells was almost absent in 2D (**Figures S6** and **S7**), and slightly increased only in RMS xenogeneic samples (about 4%). Murine PDGFRα increased particularly in xenografts derived from HT29 and MCF7 cells (**Figures 3B** and **S6**, HT29 26.02 ± 2.18% *versus* RH30 3.0 ± 1.43%), suggesting that epithelial cancer cells require much more CAFs to grow. In parallel, CXCR4 marker (also called CD184), a chemokine receptor involved in metastasis formation and poor survival of RMS patients (37), was analyzed. RMS and MCF7 cells cultured in 2D expressed a higher percentage this receptor (RH30: 86% ± 0.8; RD: 65% ± 1.2; MCF7: 61% ± 1.9) as compared to HT29 cells (11.5% ± 1.5). Although in all the cells isolated from the different xenografts CXCR4 decreased (MCF7: 10% ± 2.1, HT29: 0.15% ± 0.2), it was still higher in RMS cells (RH30: 40% ± 1.8) (**Figures 3B**, **S6** and **S7**).

Finally, cultured cells from the xenogeneic HT29 masses were positive to CAFs marker FAP protein and, to a lesser extent, to human nuclei protein (HUNU), whereas RMS cells did not express FAP and were positive only to HUNU (**Figure 3C**).

### RMS Xenogeneic Samples Highly Express ECM Remodeling Factors

**Figure 4:**
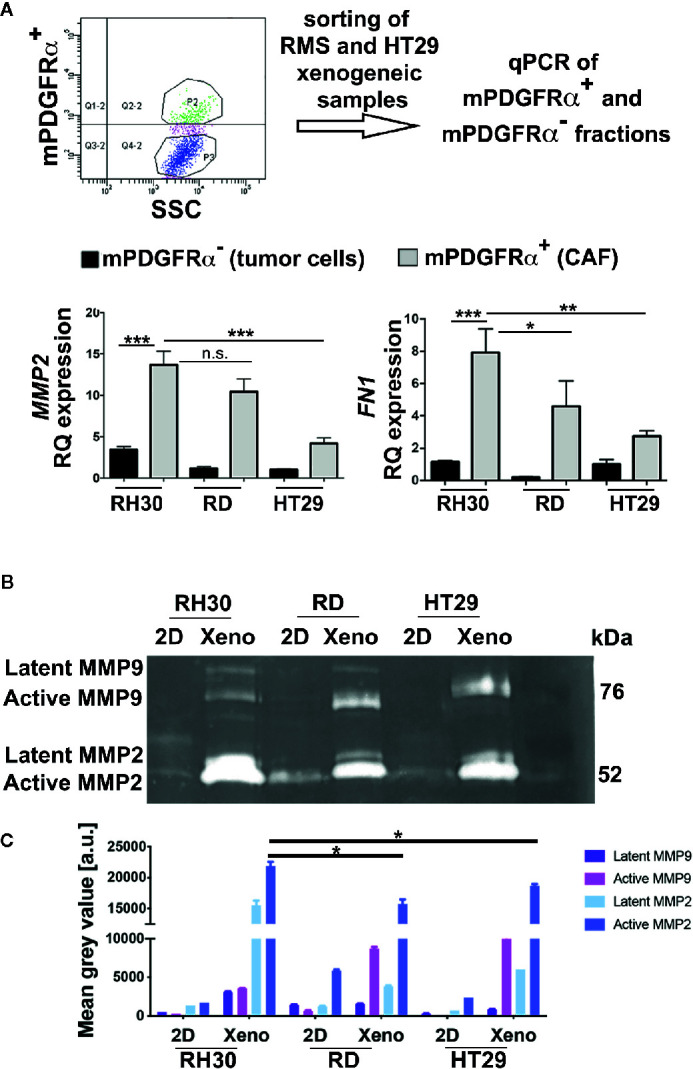
Analysis of mPDGFRα positive and negative fractions. **(A)** Upper row. The steps of the adopted experimental procedure are depicted. Lower row. Left. qPCR for *MMP2* in xenogeneic tumors (3D) of epithelial (HT29) and mesenchymal (RMS: RH30, RD cell lines) origin. The small percentage of CAFs (mPDGFRα positive cells) isolated from RH30 and RD xenogeneic samples highly expressed the gene. Right. qPCR for *FN1* in xenogeneic tumors (3D) of epithelial (HT29) and mesenchymal (RMS: RH30, RD cell lines) origin. The small percentage of CAFs (mPDGFRα positive cells) isolated from RH30 and RD xenogeneic samples highly expressed the gene. HT29 (mPDGFRα-, tumor cells) was used as calibrator. In the Figure p-value relative to two group comparison (i.e. HT29 *versus* RH30, HT29 *versus* RD) was reported (Mann-Whitney-test. *p < 0.05; ***p < 0.001; **p < 0.01). Kruskal-Wallis test was also performed (MMP2 p = 0.0067; FN1 p = 0.005). **(B)** Zymography in RH30, RD, HT29 cells grown in 2D and isolated from xenogeneic tumor masses developed in immunocompromised mice (3D). The cells cultured in 2D do not express active MMP9 and MMP2 enzymes while their 3D counterpart does (xenogeneic samples). **(C)** MMP9 and MMP2 quantification. Xenogeneic samples express higher level of the two enzymes in respect to 2D petri culture. In particular, active MMP2 is significative more expressed in RH30 xeno than HT29 xeno samples. n.s., not significant.

Since the role of CAFs is paramount in modifying the surrounding ECM, we decided to delineate the genes devoted to microenvironment remodeling in mPDGFRα-positive and -negative cell fractions (**Figure 4A**). It was striking that in the fraction of RMS xenograft cells positive to mPDGFRα (3.1 ± 1.43% for RH30 and 1.41 ± 0.9% for RD xenogeneic samples), *MMP2* was three times more expressed than in cells isolated from HT29 xenogeneic tumors (26.02 +/− 2.18%) (**Figure 4A**, **Table 4** for sorted cells), as it was fibronectin (*FN1*), one of the main structural protein of ECM. Importantly, when the proteolytic activity of MMP2 and MMP9 was assessed by zymography, RMS and HT29 cells cultured in the flat 2D conditions barely expressed both enzymes (**Figure 4B**). On the contrary, the cells isolated from the xenografts, that included also the mPDGFRα+ cells, strongly enhanced these ECM remodeling proteins and this was particularly true for MMP2 in RH30 samples (**Figures 4B, C**). MMP2 and FN1 genes were analyzed also in tumor cells cultured in 2D and extracted from the xenogeneic masses (mPDGFRα^−^ fraction). The genes were significantly more expressed in cells from the xenogeneic samples (**Figures S8A, B**). These results support the concept that cells grown in a more complex environment then the flat 2D acquire characteristics that better mimic the *in vivo* tumor microenvironment landscape, characterized by the presence of CAFs and other cells and proteins. Of note, the latent form of MMP9 was detected only in RMS cells, highlighting a more pronounced microenvironment remodeling in this type of tumors (**Figure 4C**).

**Table 4:** mPDGFRα^+^ sorted cells.

Exp N° sorting of CAFs from xenogeneic samples	XenoRH30	XenoRD	XenoHT29	XenoMCF7
#1	1.23%	0.29%	26.06%	30.64%
#2	2.85%	1.95%	23.3%	26.20%
#3	4.58%	1.1%	28.6%	25.60%
#4	3.73%	2.3%	26%	

**Figure 5:**
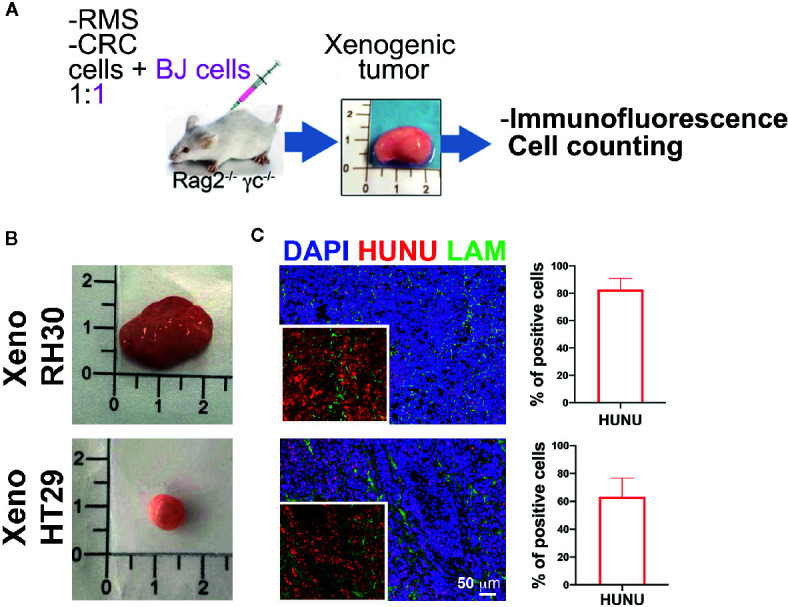
Co-injection of stromal and cancer cells. **(A)** Experimental strategy (2): cancer cells are injected without Matrigel^®^ but with stromal cells. Characterization of the xenogeneic mass and cytofluorimetric analysis of the freshly isolated cells**. (B)** Upper row. RMS mass obtained after co-injection of cancer cells (RH30 and RD) and BJ cells. Lower row. HT29 mass obtained after co-injection of HT29 cells with BJ cells. **(C)** Xenogeneic mass characterization with antibody specific for human nuclei (HUNU; red) and laminin (LAM; green). Scale bar = 50 µm. HUNU positive nuclei counting: about 80–90% of cells in xeno RH30 were positive while in xeno HT29 only about 60–70%.

In order to investigate whether stromal cells could influence the tumor growth, we developed a second approach (**Figure 5A**): we injected RH30, RD, and HT29 cells together with BJ cells, without adding Matrigel^®^, such as with cancer cells alone. Presence of human nuclei and laminin proteins characterized all formed tumors (**Figure 5C**). RMS-derived tumors were formed (**Figure 5B**) and possessed more than 80% of cells positive to HUNU. HT29 xenografts, instead, displayed about 60–70% of HUNU-positive cells and about 30% of HUNU-negative cells, most likely mPDGFRα-positive cells, in accordance with the cytofluorimetric results attained with the first approach (with Matrigel, **Figure 3**).

### ECM Structural Proteins Are Self-Secreted in RMS Cells and Xenogeneic Samples

**Figure 6:**
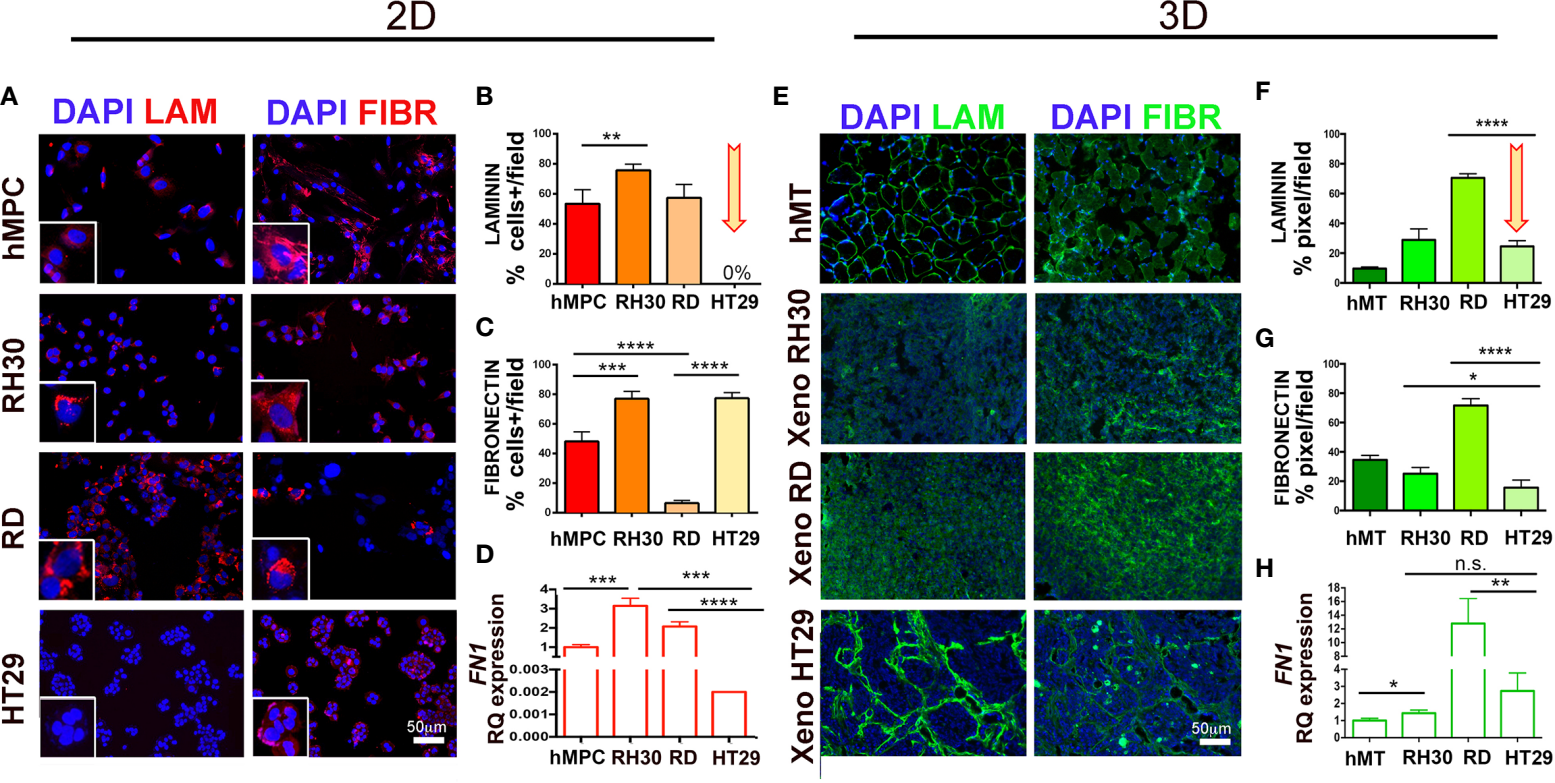
Characterization of ECM in RMS and HT29 xenogeneic tumors. **(A)** Immunofluorescence of laminin (LAM) and fibronectin (FIBR) in cells. hMPC, human muscle precursor cells. Scale bar = 50 µm. **(B)** Number of positive cells expressing laminin. HT29 cells do not express LAM (arrow). **(C)** Number of positive cells expressing fibronectin. All cell lines expressed this protein. **(D)** qPCR of *FN1* in cell lines. **(E)** Immunofluorescence of laminin (LAM) and fibronectin (FIBR) in xenogeneic samples. **(F)** Mean fluorescence intensity of laminin area in xenogeneic samples. Xeno HT29 express LAM (arrow). **(G)** Mean fluorescence intensity of fibronectin area in xenogeneic samples. hMT: human muscle tissue). **(H)** qPCR of *FN1* in xenogeneic samples. Remarkable *FN1* gene expression on tissue samples derived from RMS cell lines. In the Figure p-value relative to two group comparison (i.e. hMT *vs* RH30, HT29 *vs* RH30, HT29 *vs* RD) (Student’s t test or Mann-Whitney test) was reported. Kruskal-Wallis test was also performed (p < 0.0001). Fetal skeletal muscle was used as calibrator in qPCR tests. *p < 0.05, **p < 0.01, ***p < 0.001, ****p < 0.0001. n.s., not significant.

Since our *in vitro* and *in vivo* findings imply that RMS do not need CAFs for tumor growth, we assessed whether RMS cells are self-sufficient in producing and depositing structural and functional ECM components. To prove our hypothesis, we analyzed laminin and fibronectin expression in RH30, RD, and HT29 cell lines and xenogeneic samples. *In vitro*, RMS cells secreted both proteins, although for RH30 was more evident than for RD cells. In particular RH30 behaved similarly to human muscle precursor cells (hMPC), whereas HT29 cells produced fibronectin but not laminin (**Figures 6A–C**). The fibronectin gene expression was detected in cells cultured in 2D (at very low level in HT29 cells) and in cells extracted from xenogeneic samples (**Figures 6D, H**). Interestingly, when fibronectin expression was compared in cells from flat 2D cultures and from xenogeneic samples, a significantly higher expression was detected in the latter samples (**Figure S8B**). The same gene was highly present in cells extracted from the xenogeneic samples (**Figures 6D, H**). *In vivo*, both RMS and HT29 xenogeneic samples produced laminin and fibronectin proteins (**Figures 6E–G**). This finding supports the evidence that HT29 cells need the *in vivo* injection to express laminin. Conversely, RH30 and RD cells self-produce, already *in vitro* (2D), laminin and fibronectin. In particular, RH30 cells produce more fibronectin than RD cells; still the presence of fibronectin in RD is detected around 10%.

**Figure 7:**
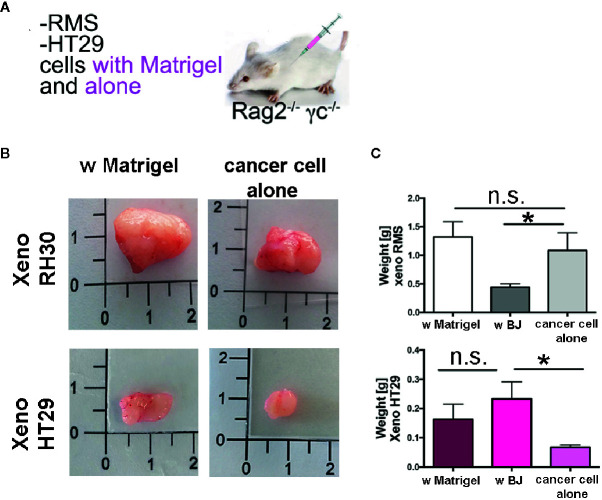
Xenogeneic samples with and without Matrigel^®^. **(A)** Experimental strategy (3): cancer cells are injected with Matrigel^®^ or alone **(B)** Pictures of the masses produced with and without matrix support of the Matrigel^®^. **(C)** Graph bars of the weight of the RMS xenogeneic masses with Matrigel^®^, with BJ and with cancer cell alone. The weight of xeno RMS did not differ with Matrigel^®^ or with cancer cell alone. **(D)** Graph bars of the weight of the HT29 xenogeneic masses with Matrigel^®^, with BJ and with cancer cell alone. HT29 xeno with BJ were heavier than with cancer cells only. HT29 only rarely gave rise to xeno masses. *p = 0.05. n.s., not significant.

Finally, to investigate whether synthesis and secretion of ECM proteins was also observed *in vivo* and used to contribute to the establishment of the tumor mass, we used the third approach (**Figure 7**). We injected cancer cells into *Rag2^−/−^γc^−/−^* mice in the presence of Matrigel^®^ or alone (**Figure 7A**). As expected, RMS cells formed big and heavy tumor masses after the two types of injections (**Figures 7B, C**, and **S9A**). The injection of HT29 cells alone, without the support of either Matrigel^®^ ECM or stromal cells (BJ fibroblasts), produced xenografts with a very low efficiency (only in the 20% of cases—2 xenografts were produced out of 10 injections), impaired also in weight and size (**Figures 7B, D**, and **S9B**). When HT29 cells were co-injected with BJ stromal cells, the weight of xenogeneic samples increased significantly to levels comparable to those obtained in the presence of Matrigel^®^. In contrast, co-injection of BJ cells with RMS RH30 cells led to development of smaller and significantly lightweight tumor masses (**Figures 7C** and **S9A**). In contrast, co-injection of BJ cells with RMS RH30 cells led to development of smaller and significantly lightweight tumor masses (**Figures 7C** and **S9A**).

## Discussion

The results of our study support the evidence that RMS cells do not need the contribution of CAFs to grow and establish tumor masses, as they produced and secreted ECM components of both structural and functional significance already in flat 2D cell culture.

Such a behavior is peculiar, since the growth and aggressiveness of many tumors, mostly of epithelial origin, are not determined only by the malignant cancer cells themselves, but also by the surrounding stroma, formed by CAFs (19). However, most of the studies on the dissection of TME have been carried out in epithelial cancers of adulthood, such as colon and breast carcinomas. In childhood cancer, such as pediatric mesenchymal sarcomas, including RMS, function, and presence of CAFs is still uncertain. The absence of specific molecular markers that allow the identification of CAFs in mesenchymal tumors makes their detection challenging and the comprehension of their role still incomplete.

Herein, as other works obtained xenogeneic masses after HT29 injection in nu/nu mice and CAF were identified (36, 38), we set up the optimal conditions to obtain the same tumor masses also in our immunocompromised mouse model. Consequently, after validating our model, we could support the new findings with our RMS xenogeneic samples. We then tailored known tools and markers for CAFs identification in epithelial tumors and applied them to analyze RMS microenvironment. In RMS few works have faced the role of CAFs. For instance, Tarnowski and colleagues demonstrated that RMS cells secrete the Macrophage Inhibitory Factor (MIF), which enhances vascularization and inhibits CAFs recruitment (39). However, the same factor promotes intestinal tumorigenesis (40) and in colon carcinoma CAFs are present contributing to chemoresistance, promoting stemness (41). Ovarian, breast and lung epithelial cancer recruit CAFs, which in turn enhance disease progression (42–44), contributing to angiogenesis and apoptotic resistance.

Fibroblasts communicate with cancer cells and other cell populations (epithelial, endothelial, inflammatory cells) through the secretion of growth factors and chemokines, becoming CAFs. Indeed, by adopting a transwell approach, we demonstrated that colon cancer HT29 cells do attract BJ stromal cells, and *vice versa*. Such a paracrine crosstalk was not observed for RMS cells.

For cell co-culture, in addition to the homogeneous cell line BJ, we used MSC from Wharton jelly, a cell population containing stromal cells, progenitor cells, fibroblasts, and stem cells (45) that better mimic the *in vivo* heterogenicity of the stromal compartment of tumors. Furthermore, since *in vitro* studies revealed that MSCs can differentiate into CAFs *via* many mechanisms such as a TGFβ1/Smad3- pathway (46–48), these cells are a good tool to recapitulate the stroma complexity. It was highlighted that HT29 spheroids stay compact with BJ cells, while RMS tend to displace them. When the supernatant of RMS spheroids was collected and used to cultivate BJ or MSC stromal cells, no changes in cell morphology, growth, viability, and motility was observed, suggesting that contact between RMS and stromal cells is somehow repulsive.

The *in vitro* results were reproduced *in vivo* by comparing xenogeneic RMS (with RH30 and RD) and CRC (with HT29) models. In summary, the results of the transwell and the spheroid experiments are consistent, because the cells do not attract each other both when seeded far away (transwell), and when put directly in contact (spheroids). We assessed a xenogeneic model which is closer to the human sample complexity in terms of crosstalk between cells, structural proteins, and growth factors. This model is also suitable to distinguish murine from human PDGFRα and, hence, host recipient CAFs from exogenous cancer cells. PDGFRα is a cell surface tyrosine kinase receptor widely expressed by many fibroblast populations including activated CAFs (49, 50), neural progenitors and pericyte cells, but also many tumor types, including glioma, prostate, and ovarian cancer (51). While HT29 cells freshly isolated from xenografts highly expressed human PDGFRα, detection in RMS xenogeneic tissues was weak. It was not important that the cell number and the time window of tumor retrieval were different between RMS and HT29 cells. It is known that each tumor type has its own intrinsic behavior. Indeed, we did not want to compare the growth rate of both tumor types (RMS and CRC), but we want to identify the CAFs population retrieved by cells incapable to grow without them, as HT29 CRC and MCF7 breast cancer cells are. Besides, we aimed at looking for CAFs in RMS xenogeneic tumors, since this model can substitute the scenario *in vivo* when human biopsies are not available for research studies, as frequently occurs in childhood cancer. We also considered αSMA and FAP as additional markers for CAFs. αSMA is member of an actin family, that plays an important role on cell motility and contractility; it stimulates myofibroblast contractility upon wound healing. FAP, instead, is a type II integral membrane serine protease, upregulated in epithelial carcinomas and tumor activated fibroblasts (52). As myofibroblasts are numerous in TME, αSMA becomes a specific marker to identify CAFs (53). Nonetheless, RH30 and RD cells express αSMA and we could not use this marker to identify CAFs in RMS cell samples. In contrast, when anti-FAP antibody was used in RMS and HT29-derived xenogeneic samples, FAP was detected only in the latter, mainly in cells of murine origin, therefore supporting the concept that RMS cells, unlike HT29 epithelial cancer cells, do not need fibroblasts to build and remodel their own ECM and to grow. Beyond the regulation of inflammation and wound healing (54, 55), fibroblast functions include the deposition of ECM proteins such as fibronectin, collagen, and laminin (55, 56). In addition, they are also an important source of ECM-degrading proteases such as matrix metalloproteinases (MMPs), which points out their crucial role in regulating ECM homeostasis and turnover (57).

PDGFR is a well-recognized CAF marker (11). Strikingly, the small percentage of PDGFRα positive cells isolated from RMS samples express homogeneously *MMP2* and *FN1* genes, as the murine PDGFRα positive cells isolated from HT29 samples. We can speculate that the homogeneous behavior of the few stromal cells in RMS xenografts is similar to the CAFs found in HT29 xenografts. As clearly shown in the co-culture of RH30-GFP spheroids and BJ (in **Supplementary Material S3**), the cells of the spheroids and the fibroblasts do not communicate with each other: spheroids disaggregate as long as fibroblasts escape from them. We can postulate that RMS cells and fibroblasts act independently. Indeed, given the complexity of the tumor environment, we cannot be sure that RMS cells are able to induce the expression of the above genes in fibroblasts. Further investigation will shed light on this aspect. We investigated the expression of human PDGFRα because it is recognized as marker of tumor RMS cells, while the variant β (human PDGFRβ) was primarily detected in vascular stroma and it has been shown to be more related to wound healing and leukocyte differentiation (22). In our hands, the RMS cells cultured in 2D did not express this marker and it was interesting to see how the 3D environment induced in RH30 cells the production of the surface protein. This also confirmed the autocrine mechanism previously described in RMS cells (58), since these cells have been shown to express a lot of growth factors including the majority of PDGFR ligands. In addition, zymography confirmed the stronger secretion of MMP2 activity exclusively in 3D-growing cancer cells, providing the evidence that the 3D environment enhances the expression of genes and proteins related to the ECM. Indeed, when Matrigel^®^ was used to support RMS cells growth and expansion *in vivo*, xenogeneic masses did not change in size and morphology compared to those obtained in the absence of it, proving the ability of RMS cells to generate a new ECM by themselves. When BJ stromal cells were co-injected, instead, RMS tumors were reduced in weight tough maintaining a similar growth rate. In summary, murine CAFs were few and played a less important role in growth of RMS than epithelial cancer cells. The activated stromal cells are the major depositors of ECM components in the TME but the self-production of extracellular matrix proteins, such as fibronectin, laminin, and MMPs proved to be paramount for the growth of RMS xenografts. Of note, RMS cells share features with myoblast like cells and are similar to healthy human muscle cells with respect to fibronectin and laminin production. The differences on fibronectin production that have been found between RH30 and RD cells can be related to the different origin of the cells: RH30 cells belong to the more aggressive alveolar RMS subtype, whereas RD to the less aggressive embryonal RMS.

Mesenchymal RMS cancer cells resemble healthy muscle cells, one of the most highly regenerating cell population in the human body. This characteristic, together with their pathological transformation, could be one of the reasons explaining their incredible capability to self-sustain and organize their growth.

In conclusion, further investigations of the microenvironment are paramount to understand which elements are crucial for RMS tumorigenesis. Injection with CAFs should be performed to investigate whether RMS cells will still recruit CAFs from the host since it has already been demonstrated that xenogeneic epithelial cancer are more aggressive when cancer cell lines are co-injected with CAFs (59). From the literature there are important examples on how surrounding environment influences tumor fate. In sarcomas, a recent work demonstrated how a collagen matrix crosslinker modifies the mechanical and chemical properties of the microenvironment, accelerating tumor cells motility (60). In perspective, ECM proteins in RMS can exert a pleiotropic effect in the milieu and their role on disease progression could be the new discovered target to block tumor growth in young patients.

## Data Availability Statement

The raw data supporting the conclusions of this article will be made available by the authors, without undue reservation.

## Ethics Statement

The animal study was reviewed and approved by CEASA, protocol 304/2017.

## Author Contributions

SD’A: conception and design, collection and assembling of the data, data analysis and interpretation, manuscript writing. LT, MS, CF, ER, SP, FF, CB, PR, PG: collection and assembling of the data. PB, SA, RG: analysis and interpretation. MP: conception and design, data assembling, analysis and interpretation, manuscript writing. All authors contributed to the article and approved the submitted version.

## Funding

This work has been supported by Progetto di Ateneo 2016 (Budget Integrato per la Ricerca dei Dipartimenti), Padova University and by Assocazione Puzzle, Padova, Italy. MP was funded by University of Padova, Grant number GRIC15AIPF, Assegno di Ricerca Senior.

## Conflict of Interest

The authors declare that the research was conducted in the absence of any commercial or financial relationships that could be construed as a potential conflict of interest.

## References

[1] Kim JR Yoon HM Koh K-N Jung AY Cho YA Lee JS Rhabdomyosarcoma in Children and Adolescents: Patterns and Risk Factors of Distant Metastasis. Pediatr Imaging (2017) 209(2):406–16. 10.2214/AJR.16.17466 28590782

[2] Hinson ARP Jones R Crose LE Belyea BC Barr FG Linardic CM Human rhabdomyosarcoma cell lines for rhabdomyosarcoma research: utility and pitfalls. Front Oncol (2013) 3(July):183. 10.3389/fonc.2013.00183 23882450 PMC3713458

[3] Saab R Spunt SL Skapek SX Myogenesis and Rhabdomyosarcoma: The Jekyll and Hyde of Skeletal Muscle. In: Current Topics in Developmental Biology, vol. 94 (2011). p. 197–234. 10.1016/B978-0-12-380916-2.00007-3 21295688

[4] Charytonowicz E Cordon-Cardo C Matushansky I Ziman M Alveolar rhabdomyosarcoma: Is the cell of origin a mesenchymal stem cell? Cancer Lett (2009) 279(2):126–36. 10.1016/j.canlet.2008.09.039 19008039

[5] Monti E Fanzani A Uncovering metabolism in rhabdomyosarcoma. Cell Cycle (2016) 15(2):184–95. 10.1080/15384101.2015.1071746 PMC4825834 26209235

[6] Correia AL Bissell MJ The tumor microenvironment is a dominant force in multidrug resistance. Drug Resist Updat (2012) 15(0):39–49. 10.1016/j.drup.2012.01.006 22335920 PMC3658318

[7] Valkenburg KC De Groot AE Pienta KJ Targeting the tumour stroma to improve cancer therapy. Nat Rev Clin Oncol (2018) 15(6):366–81. 10.1038/s41571-018-0007-1 PMC5960434 29651130

[8] Egeblad M Nakasone ES Werb Z Tumors as organs: complex tissues that interface with the entire organism. Dev Cell (2010) 18(6):884–901. 10.1016/j.devcel.2010.05.012 20627072 PMC2905377

[9] Wu J Liang C Chen M Su W Association between tumor-stroma ratio and prognosis in solid tumor patients: A systematic review and meta-analysis. Oncotarget (2016) 7(42):68954–65. 10.18632/oncotarget.12135 PMC5356603 27661111

[10] Lindner D Zietsch C Becher PM Schulze K Schultheiss HP Tschöpe C Differential expression of matrix metalloproteases in human fibroblasts with different origins. Biochem Res Int (2012) 2012:1–10. 10.1155/2012/875742 PMC3303709 22500233

[11] Kalluri R Zeisberg M Fibroblasts in cancer. Nat Rev Cancer (2006) 6(5):392–401. 10.1038/nrc1877 16572188

[12] Li B Wang JH-C. Fibroblasts and Myofibroblasts in Wound Healing: Force Generation and Measurement. J Tissue Viability (2011) 20(4):108–20. 10.1016/j.jtv.2009.11.004 PMC2891362 19995679

[13] Dvorak HF Tumors: wounds that do not heal. Redux Cancer Immunol Res (2015) 3(1):1–11. 10.1158/2326-6066.CIR-14-0209 25568067 PMC4288010

[14] Eble JA Niland S The extracellular matrix in tumor progression and metastasis. Clin Exp Metastasis Springer Netherlands (2019) 36:171–98. 10.1007/s10585-019-09966-1 30972526

[15] Shimoda M Jackson HW Khokha R Tumor suppression by stromal TIMPs. Mol Cell Oncol (2016) 3(3):e975082. 10.4161/23723556.2014.975082 27314104 PMC4909426

[16] Lambrechts D Wauters E Boeckx B Aibar S Nittner D Burton O Phenotype molding of stromal cells in the lung tumor microenvironment. Nat Med (2018) 24(8):1277–89. 10.1038/s41591-018-0096-5 29988129

[17] Su S Chen J Yao H Liu J Yu S Lao L CD10+GPR77+ Cancer-Associated Fibroblasts Promote Cancer Formation and Chemoresistance by Sustaining Cancer Stemness. Cell (2018) 172(4):841–56.e16. 29395328 10.1016/j.cell.2018.01.009

[18] Puré E Blomberg R Pro-tumorigenic roles of fibroblast activation protein in cancer: back to the basics. Oncogene (2018) 37(32):4343–57. 10.1038/s41388-018-0275-3 PMC6092565 29720723

[19] Kalluri R The biology and function of fibroblasts in cancer. Nat Rev Cancer (2016) 16(9):582–98. 10.1038/nrc.2016.73 27550820

[20] Roche J The Epithelial-to-Mesenchymal Transition in Cancer. Cancers (Basel) (2018) 10(2):52. 10.3390/cancers10020052 PMC5836084 29462906

[21] Ehnman M Chaabane W Haglund F Tsagkozis P The Tumor Microenvironment of Pediatric Sarcoma: Mesenchymal Mechanisms Regulating Cell Migration and Metastasis. Curr Oncol Rep (2019) 21(10):90. 10.1007/s11912-019-0839-6 31418125 PMC6695368

[22] Ehnman M Missiaglia E Folestad E Selfe J Strell C Thway K Distinct effects of ligand-induced PDGFRα and PDGFRβ signaling in the human rhabdomyosarcoma tumor cell and stroma cell compartments. Cancer Res (2013) 73(7):2139–49. 10.1158/0008-5472.CAN-12-1646 PMC3672973 23338608

[23] Saggioro M D’Angelo E Bisogno G Agostini M Pozzobon M Carcinoma and Sarcoma Microenvironment at a Glance: Where We Are. Front Oncol (2020) 10(March):1–9. 10.3389/fonc.2020.00076 32195166 PMC7063801

[24] Nyga A Cheema U Loizidou M 3D tumour models: Novel in vitro approaches to cancer studies. J Cell Commun Signal (2011) 5(3):239–48. 10.1007/s12079-011-0132-4 PMC3145874 21499821

[25] Franzin C Piccoli M Urbani L Biz C Gamba P De Coppi P Isolation and Expansion of Muscle Precursor Cells from Human Skeletal Muscle Biopsies. Methods Mol Biol (2016) 1341:257–84. 10.1007/7651_2016_321 27032940

[26] Pozzobon M Saggioro M D’Agostino S Bisogno G Muraca M Gamba P Alveolar Rhabdomyosarcoma Decellularization. Methods Mol Biol (2017) 1341:257–84. 10.1007/7651_2017_45 28540560

[27] Tajhya RB Patel RS Beeton C Detection of matrix metalloproteinases by zymography. Methods Mol Biol (2017) 1579:231–44. 10.1007/978-1-4939-6863-3_12 PMC5465868 28299740

[28] Frankowski H Gu Y-H Heo JH Milner R del Zoppo GJ Use of Gel Zymography to Examine Matrix Metalloproteinase (Gelatinase) Expression in Brain Tissue or in Primary Glial Cultures. Methods Mol Biol (2012) 814(1):1–11. 10.1007/978-1-61779-452-0_15 PMC3670093 22144310

[29] Schindelin J Arganda-Carrera I Frise E Verena K Mark L Tobias P Fiji - an Open platform for biological image analysis. Nat Methods (2012) 9(7):676–82. 10.1038/nmeth.2019 PMC3855844 22743772

[30] Kinugasa Y Matsui T Takakura N CD44 expressed on cancer-associated fibroblasts is a functional molecule supporting the stemness and drug resistance of malignant cancer cells in the tumor microenvironment. Stem Cells (2014) 32(1):145–56. 10.1002/stem.1556 24395741

[31] Dolznig H Walzl A Kramer N Rosner M Garin-Chesa P Hengstschläger M Organotypic spheroid cultures to study tumor-stroma interaction during cancer development. Drug Discovery Today Dis Model (2011) 8(2–3):113–9. 10.1016/j.ddmod.2011.06.003

[32] Millán-Rivero JE Nadal-Nicolás FM García-Bernal D Sobrado-Calvo P Blanquer M Moraleda JM Human Wharton’s jelly mesenchymal stem cells protect axotomized rat retinal ganglion cells via secretion of anti-inflammatory and neurotrophic factors. Sci Rep (2018) 8(1):1–14. 10.1038/s41598-018-34527-z 30389962 PMC6214908

[33] Kannaiyan J Narayanan S MP Pandey A Acute toxicity study of Mesenchymal Stromal cells derived from Wharton’s Jelly in mouse by intravenous and subcutaneous route. Int J Res Dev Pharm Life Sci (2017) 6(5):2748–56. 10.21276/IJRDPL.2278-0238.2017.6(5).2748-2756

[34] Marino L Castaldi MA Rosamilio R Ragni E Vitolo R Fulgione C Mesenchymal stem cells from the Wharton’s jelly of the human umbilical cord: Biological properties and therapeutic potential. Int J Stem Cells (2019) 12(2):218–26. 10.15283/ijsc18034 PMC6657936 31022994

[35] Özer E Alveolar rhabdomyosarcoma. Pathol Outlines.com Web (2017). Available from: https://www.pathologyoutlines.com/topic/softtissuealvrhabdo.html.

[36] Szot C Saha S Zhang XM Zhu Z Hilton MB Morris K Tumor stroma–targeted antibody-drug conjugate triggers localized anticancer drug release. J Clin Invest (2018) 128(7):2927–43. 10.1172/JCI120481 PMC6025988 29863500

[37] Vela M Bueno D González-Navarro P Brito A Fernández L Escudero A Anti-CXCR4 antibody combined with activated and expanded natural killer cells for sarcoma immunotherapy. Front Immunol (2019) 10. 10.3389/fimmu.2019.01814 PMC6688426 31428099

[38] Tentler JJ Bradshaw-Pierce EL Serkova NJ Hasebroock KM Pitts TM Diamond JR Assessment of the in vivo antitumor effects of ENMD-2076, a novel multitargeted kinase inhibitor, against primary and cell line-derived human colorectal cancer xenograft models. Clin Cancer Res (2010) 16(11):2989–98. 10.1158/1078-0432.CCR-10-0325 PMC3928713 20406842

[39] Tarnowski M Grymula K Liu R Tarnowska J Drukala J Ratajczak J Macrophage migration inhibitory factor is secreted by rhabdomyosarcoma cells, modulates tumor metastasis by binding to CXCR4 and CXCR7 receptors and inhibits recruitment of cancer-associated fibroblasts. Mol Cancer Res (2010) 8(10):1328–43. 10.1158/1541-7786.MCR-10-0288 PMC2974061 20861157

[40] He XX Chen K Yang J Li XY Gan HY Liu CY Macrophage migration inhibitory factor promotes colorectal cancer. Mol Med (2009) 15(1–2):1–10. 10.2119/molmed.2008.00107 19009023 PMC2581606

[41] Ren J Ding L Zhang D Shi G Xu Q Shen S Carcinoma-associated fibroblasts promote the stemness and chemoresistance of colorectal cancer by transferring exosomal lncRNA H19. Theranostics (2018) 8(14):3932–48. 10.7150/thno.25541 PMC6071523 30083271

[42] Schauer IG Sood AK Mok S Liu J Cancer-associated fibroblasts and their putative role in potentiating the initiation and development of epithelial ovarian cancer. Neoplasia (2011) 13(5):393–405. 10.1593/neo.101720 21532880 PMC3084616

[43] Wu HJ Hao M Yeo SK Guan JL FAK signaling in cancer-associated fibroblasts promotes breast cancer cell migration and metastasis by exosomal miRNAs-mediated intercellular communication. Oncogene (2020) 39(12):2539–49. 10.1038/s41388-020-1162-2 PMC7310603 31988451

[44] Zhou Z Zhou Q Wu X Xu S Hu X Tao X VCAM-1 secreted from cancer-associated fibroblasts enhances the growth and invasion of lung cancer cells through AKT and MAPK signaling. Cancer Lett (2019) 473:62–73. 10.1016/j.canlet.2019.12.039 31904479

[45] Brown T The gap between the physiologycal and therapeutic roles of mesenchymal stem cells. Harv Bus Rev (2008) 86(6):84–92. 18605031

[46] Mishra PJ Mishra PJ Humeniuk R Medina DJ Alexe G Mesirov JP Carcinoma-Associated Fibroblast – Like Differentiation of Human Mesenchymal Stem Cells. Cancer Res (2008) 68(11):4331–9. 10.1158/0008-5472.CAN-08-0943 PMC2725025 18519693

[47] Alt E Welte G Li J Hennessy BT Devarajan E Krishnappa S Adipose tissue-derived stem cells differentiate into carcinoma-associated fibroblast-like cells under the influence of tumor-derived factors. Anal Cell Pathol (2010) 33(2):61–79. 10.1155/2010/695162 PMC4605656 20978328

[48] Sugihara H Ishimoto T Yasuda T Izumi D Eto K Sawayama H Cancer-associated fibroblast-derived CXCL12 causes tumor progression in adenocarcinoma of the esophagogastric junction. Med Oncol (2015) 32(6):168. 10.1007/s12032-015-0618-7 25920609

[49] Erez N Truitt M Olson P Hanahan D Cancer-Associated Fibroblasts Are Activated in Incipient Neoplasia to Orchestrate Tumor-Promoting Inflammation in an NF-κB-Dependent Manner. Cancer Cell (2010) 17(2):135–47. 10.1016/j.ccr.2009.12.041 20138012

[50] Higashino N Koma YI Hosono M Takase N Okamoto M Kodaira H Fibroblast activation protein-positive fibroblasts promote tumor progression through secretion of CCL2 and interleukin-6 in esophageal squamous cell carcinoma. Lab Invest (2019) 99(6):777–92. 10.1038/s41374-018-0185-6 30683902

[51] Heldin C-H Heldin C-H Westermark B Andrae J Gallini R Betsholtz C Targeting the PDGF signaling pathway in tumor treatment. Cell Commun Signal (2013) 11(1):97. 10.1186/1478-811X-11-97 24359404 PMC3878225

[52] Isella C Terrasi A Bellomo SE Petti C Galatola G Muratore A Stromal Contribution To Colorectal Cancer Transcript. Nat Genet (2015) 47(4):312–9. 10.1038/ng.3224 25706627

[53] Micallef L Vedrenne N Billet F Coulomb B Darby IA Desmoulière A The myofibroblast, multiple origins for major roles in normal and pathological tissue repair. Fibrogenes Tissue Repair (2012) 5:1–5. 10.1186/1755-1536-5-S1-S5 PMC3368789 23259712

[54] Tomasek JJ Gabbiani G Hinz B Chaponnier C Brown RA Myofibroblasts and mechano- regulation of connective tissue remodelling. Nat Rev Mol Cell Biol (2002) 3(5):349–63. 10.1038/nrm809 11988769

[55] Parsonage G Filer AD Haworth O Nash GB Rainger GE Salmon M A stromal address code defined by fibroblasts. Trends Immunol (2005) 26(3):150–6. 10.1016/j.it.2004.11.014 PMC3121558 15745857

[56] Chang HY Chi JT Dudoit S Bondre C Van De Rijn M Botstein D Diversity, topographic differentiation, and positional memory in human fibroblasts. Proc Natl Acad Sci USA (2002) 99(20):12877–82. 10.1073/pnas.162488599 PMC130553 12297622

[57] Simian M Hirai Y Navre M Werb E Lochter A Bissel MJ The interplay of matrix metalloproteinases, morphogens and growth factors is necessary for branching of mammary epithelial cells. Development (2008) 23(1):1–7. 10.1242/dev.128.16.3117 PMC2785713 11688561

[58] Östman A PDGF receptors-mediators of autocrine tumor growth and regulators of tumor vasculature and stroma. Cytokine Growth Factor Rev (2004) 15(4):275–86. 10.1016/j.cytogfr.2004.03.002 15207817

[59] Orimo A Gupta PB Sgroi DC Arenzana-Seisdedos F Delaunay T Naeem R Stromal fibroblasts present in invasive human breast carcinomas promote tumor growth and angiogenesis through elevated SDF-1/CXCL12 secretion. Cell (2005) 121(3):335–48. 10.1016/j.cell.2005.02.034 15882617

[60] Lewis DM Pruitt H Jain N Ciccaglione M Michael McCaffery J Xia Z A feedback loop between hypoxia and matrix stress relaxation increases oxygen-axis migration and metastasis in sarcoma. Cancer Res (2019) 79(8):1981–95. 10.1158/0008-5472.CAN-18-1984 PMC6727644 30777851

