# Supplementary material for: Rhabdomyosarcoma Cells Produce Their Own Extracellular Matrix With Minimal Involvement of Cancer-Associated Fibroblasts: A Preliminary Study
Source: Front Oncol. 2021 Jan 29;10:600980. doi: 10.3389/fonc.2020.600980 (PMC7878542; doi:10.3389/fonc.2020.600980)
Supplement: Supplementary file 1 [file DataSheet_1.pdf]

D’Agostino et al. Supplementary Material

1.1 Supplementary Figures

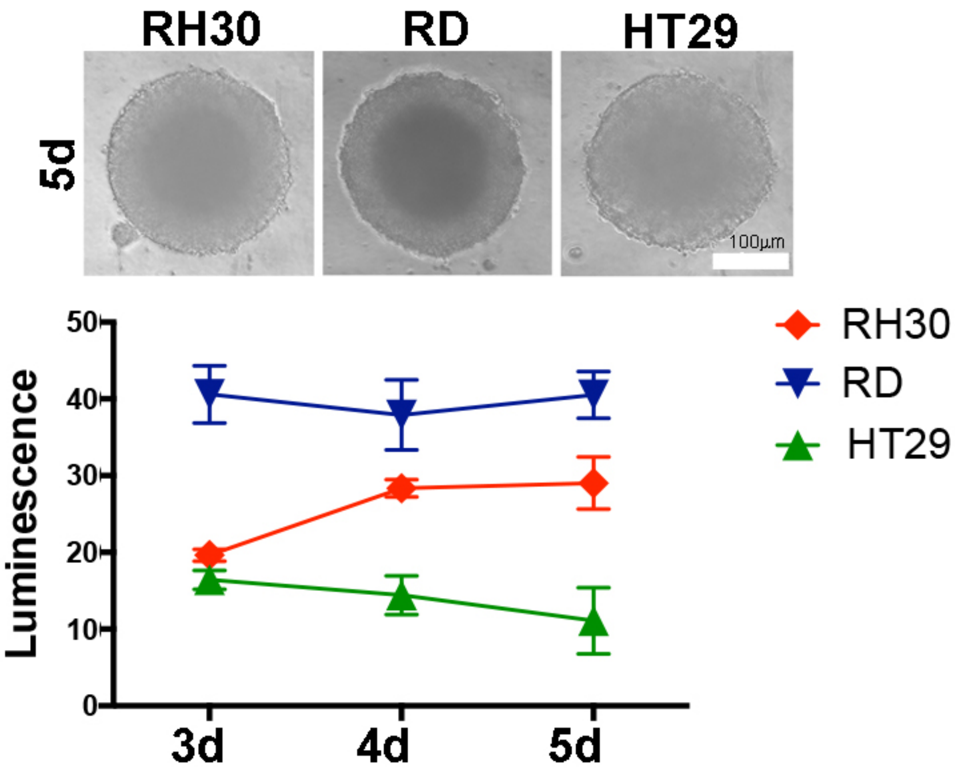

Figure S1. Spheroid viability test.

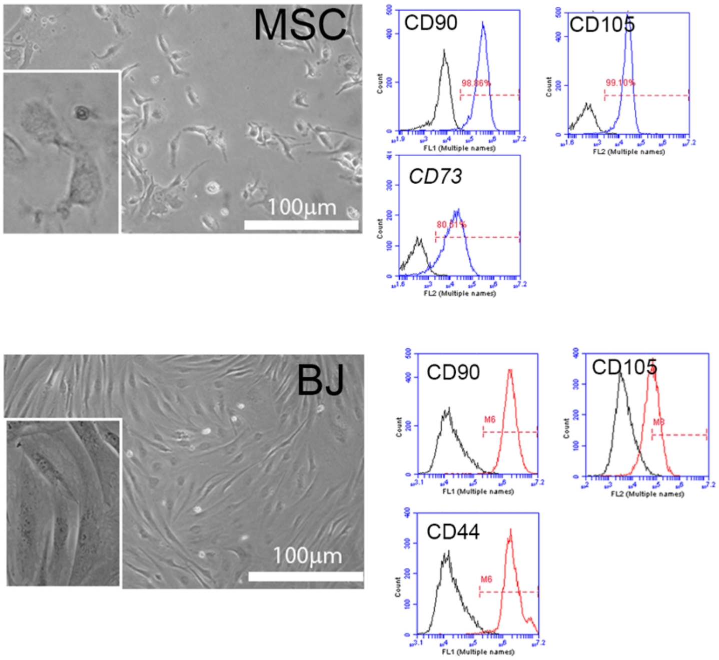

Figure S2. Wharton Jelly MSC and BJ characterization.

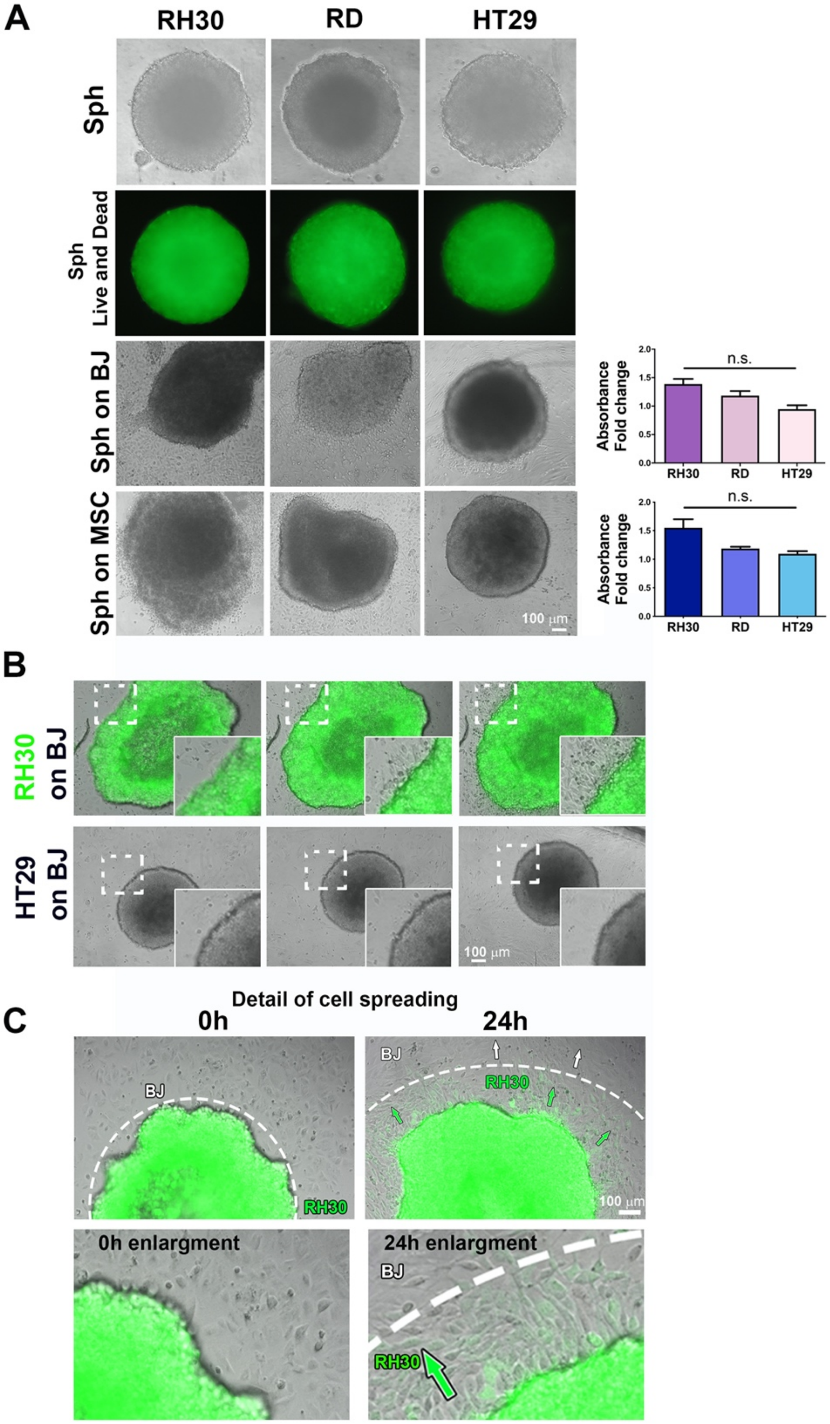

Figure S3. Spheroids on stromal cells. (A) Spheroids without layer (first line), live and dead of spheroids cultured alone (second line), spheroids on the top of the stromal cells after 48h (third and fourth line, left). Toxicity evaluation with LDH (third and fourth line, right) using spheroid supernatant as control. **(B)** First line: time lapse of RH30 spheroids on BJ layer. After 12h and 24h it can be appreciated that the cell contact between the two cell types gives rise to cell spreading of RH30 and BJ. Second line: HT29 spheroids stay compact when in contact with BJ cells. **(C) First line and second line.** Detail of the spreading of the RH30 spheroid when put on the top of BJ. It can be appreciated the

movement of RH30 (and BJ) cells during time. After 24h, both RH30 and BJ move from the spheroid border.

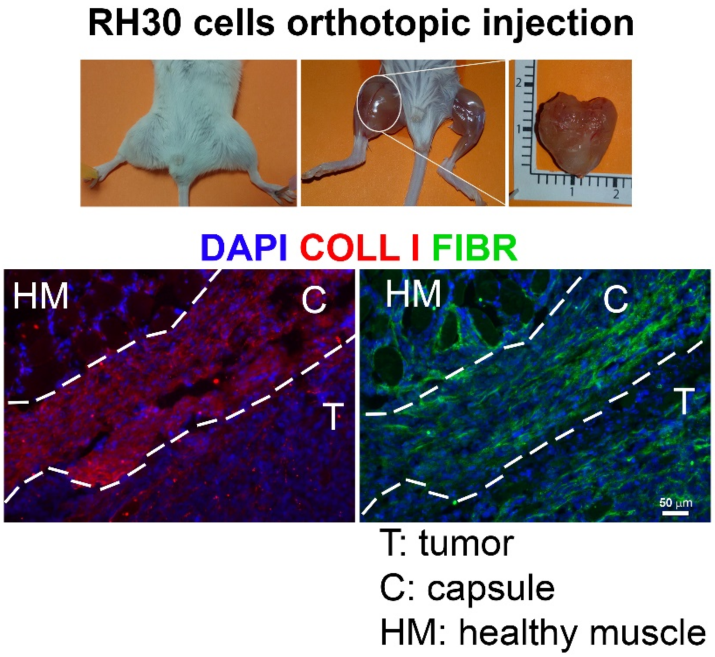

Figure S4. RMS orthotopic injection. First line. Gross appearance of the RMS injection directly in muscle. **Second line**. Immunofluorescence of the RH30 xenograft with part of healthy muscle. The capsule surrounding the tumors was full of collagen and fibronectin. The wrapping of the healthy muscle with the tumor cells made the analysis difficult and inconsistent. Scale bar: 50µm.

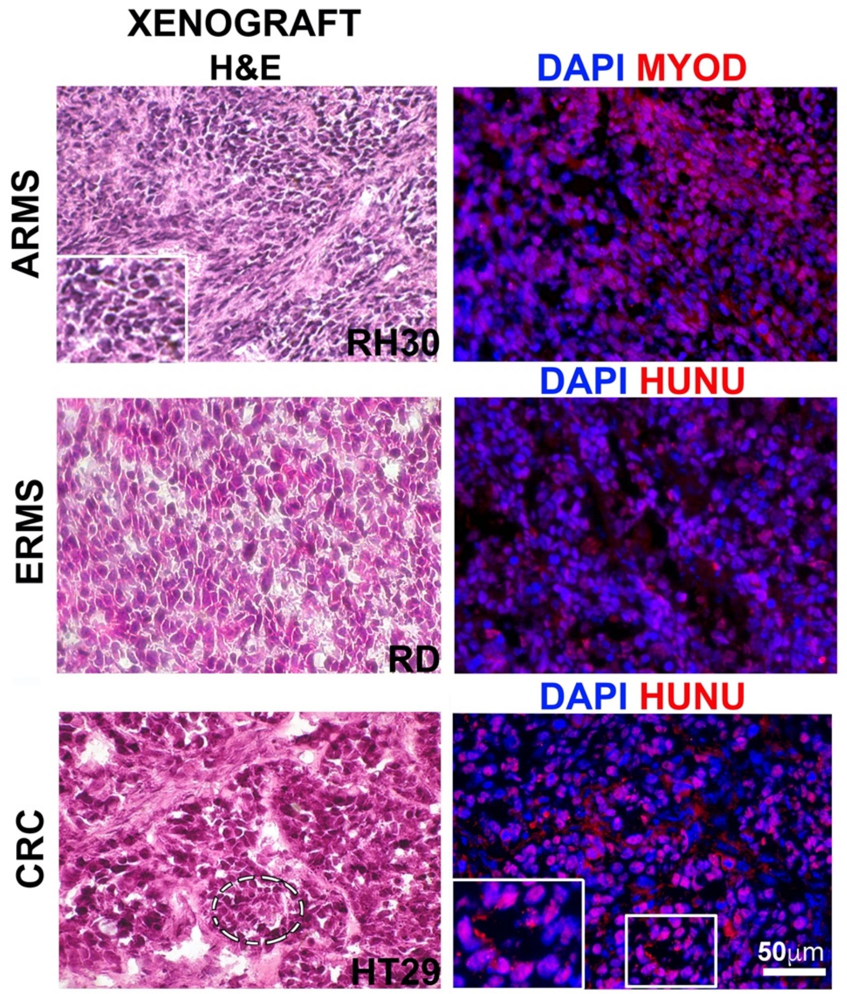

Figure S5. Histological analysis of the xenografts. First line, left, xenograft from RH30 cells, in which the thin fibrous septae separating the cellular nests can be appreciated (first line, in the middle). MYOD staining (first line, right) underlined the myogenic signature of ARMS-derived samples. (Image for the human biopsies comparable with the xenograft can be found: ARMS: https://www.pathologyoutlines.com/topic/softtissuealvrhabdo.html**.** Image: http://www.pathologyoutlines.com/images/softtissue/06_35G.jpg **. Second line**, left. Spindle and round cells are characteristic to human ERMS samples, as evident in the xnograft from RD cells (second line, in the middle). Immunofluorescence for the Human Nuclei antigen (HUNU) highlights the strong prevalence of human cells in our xenograft samples (right). (Image for the human biopsies comparable with the xenograft can be found: ERMS: https://www.pathologyoutlines.com/topic/softtissueembryonalrhabdo.html **.** Image: http://www.pathologyoutlines.com/wick/softtissue/rhabdomyosarcomaembryonaltyperetroorbitalmicro1.jpg)**. Third line**, alveoli with necrotic debris are visible in HT29 xenograft samples (white circle). The picture on the right underlines that the same alveolar structures are mostly positive for the HUNU antigen, and some of the cells are of murine origin. (Image for the human biopsies comparable with the xenograft can be found: CRC: https://www.pathologyoutlines.com/topic/colontumoradenocarcinoma.html Image: https://webpath.med.utah.edu/GIHTML/GI120.html)

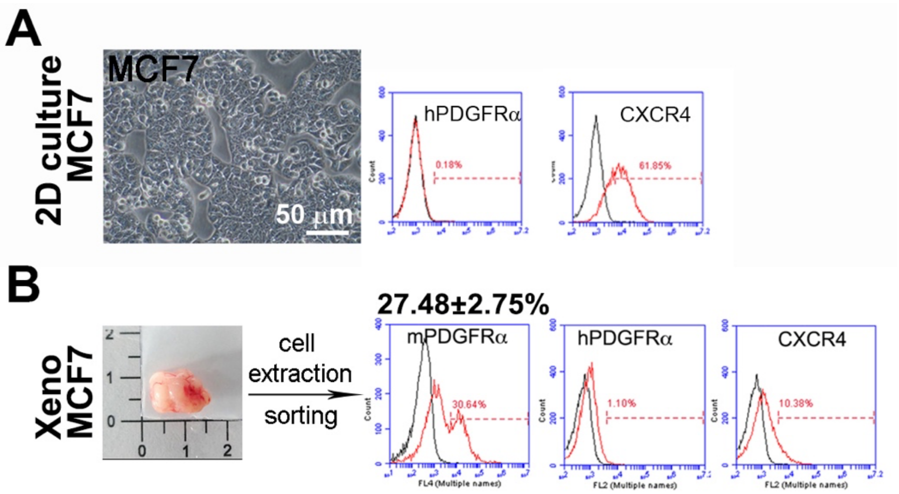

Figure S6. MCF7 cells in 2D and 3D. (A). MCF7 bright field, human PDGFRα was negative while CXCR4 was highly positive in 2D culture. (**B).** Flow cytometric based analysis of the cells isolated from MCF7-derived xenograft. Murine human PDGFRa gave positive signal, while the human counterpart was negative and CXCR4 strongly decreased.

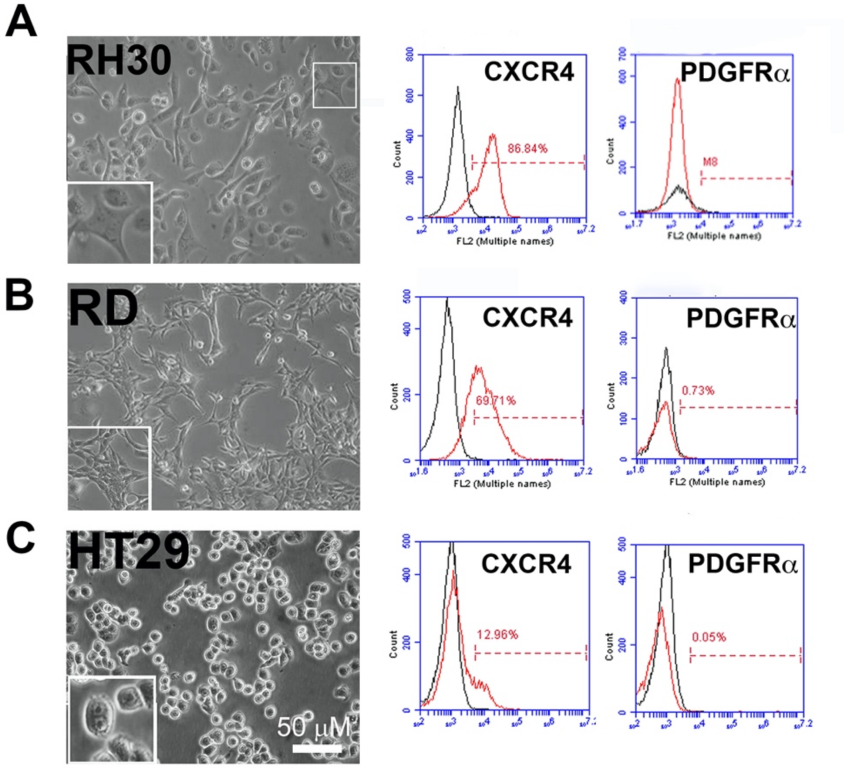

Figure S7. Expression of CXCR4 and PDGFRα for cells cultured in 2D. (A) Bright field image of RH30 cells, highly expressing CXCR4 but negative for PDGFRα. **(B)** Bright field image of RD cells, highly expressing CXCR4 but almost negative for PDGFRα. **(C)** Bright field image of HT29 cells, expressing CXCR4 at lower level in respect to RMS cells, but still negative for PDGFRα.

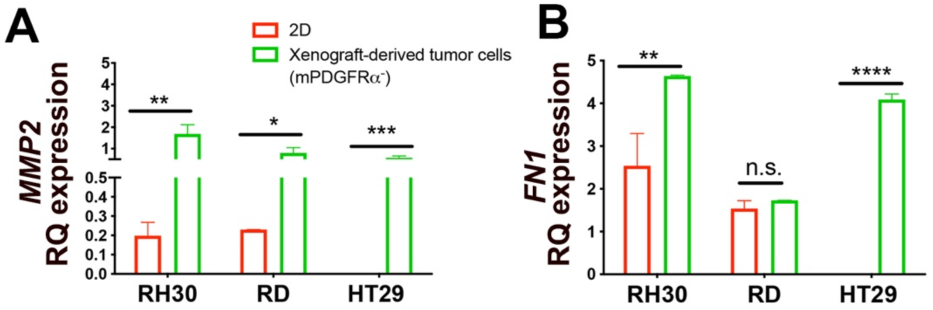

Figure S8. Expression of MMP2 and FN1 in 2D and xenogeneic derived cells. (A) *MMP2* expression in cells from 2D cultures and extracted from 3D samples (xenogeneic samples). The gene is highly expressed in the latter samples. **(B)** Fibronectin (*FN1)* in cells extracted from 3D samples (xenogeneic samples) is significantly higher in respect to 2D cells.

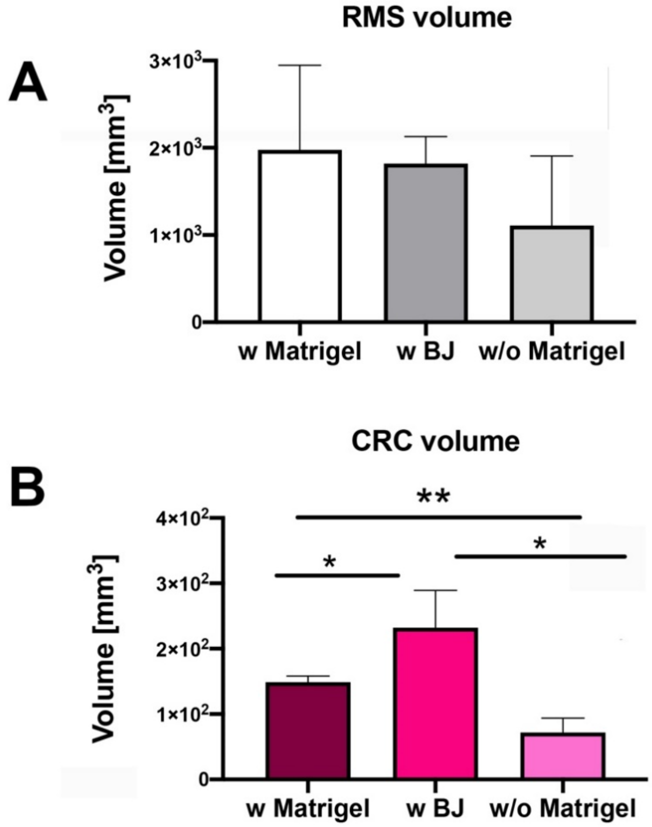

Figure S9. Volume of the xenografts. (A) For RMS xenografts no significant difference in volume was detected. **(B)** HT29 xenografts produced with BJ were bigger than samples with and without Matrigel.
